# Dynamic Modelling and Experimental Characterization of a Self-Powered Structural Health-Monitoring System with MFC Piezoelectric Patches †

**DOI:** 10.3390/s20040950

**Published:** 2020-02-11

**Authors:** Gianpietro Di Rito, Mario Rosario Chiarelli, Benedetto Luciano

**Affiliations:** 1 Dipartimento di Ingegneria Civile ed Industriale-Università di Pisa, 56122 Pisa, Italy; mario.rosario.chiarelli@unipi.it; 2 AESIS srl, 56017 San Giuliano Terme (PI), Italy; b.luciano@aesis-studio.it

**Keywords:** structural monitoring, health-management, piezoelectricity, actuators, sensors, energy harvesters, modeling, testing

## Abstract

The paper deals with theoretical and experimental studies for the development of a self-powered structural health monitoring (SHM) system using macro-fiber composite (MFC) patches. The basic idea is to integrate the actuation, sensing, and energy harvesting capabilities of the MFC patches in a SHM system operating in different regimes. As an example, during flight, under the effects of normal structural vibrations, the patches can work as energy harvesters by maintaining or restoring the battery charge of the stand-by SHM electronic board; on the other hand, if relevant/abnormal loadings are applied, or if local faults produce a noticeable stiffness variation of the monitored component, the patches can act as sensors for the power-up SHM board. During maintenance, the patches can then work as actuators, to stress the structure with pre-defined load profiles, as well as sensors, to monitor the structural response. In this paper, the investigation, based on the electromechanical impedance technique, is carried out on a system prototype made of a cantilevered composite laminate with six MFC patches. A high-fidelity nonlinear model of the system, including the piezoelectric hysteresis of the patches and three vibration modes of the laminate beam, is presented and validated with experiments. The results support the potential feasibility of the system, pointing out that the energy storage can be used for recharging a 3V-65mAh Li-ion battery, suitable for low-power electronic boards. The model is finally used to characterize a condition-monitoring algorithm in terms of false alarms rejection and vulnerability to dormant faults, by simulating built-in tests to be performed during maintenance.

## 1. Introduction

**Figure 1:**
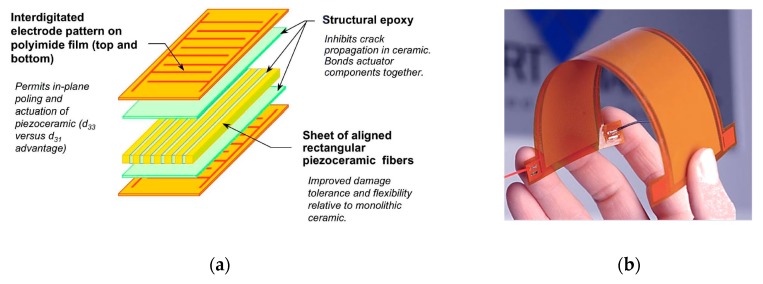
Macro-fiber composite (MFC) piezoelectric devices [27]: (**a**) structural arrangement; (**b**) patch layout.

Piezoelectric materials are widely used in a variety of aerospace applications in both sensing and low-power actuation devices, and many research works discuss the use of such materials as actuators, sensors, or energy harvesters. An extensive literature documents the applications of piezoelectric actuators, with special focus on noise abatement and control [1,2,3,4], the design of smart mechanical systems [5,6], the development of self-shaping structures with vibration control capabilities [7,8,9], or the design of smart devices for aerodynamic flow control [10,11,12,13,14,15,16]. Important research works also focus on the study of the energy harvesting capability of piezoelectric materials [17,18,19,20], as well as on their application as sensors in structural health monitoring (SHM) systems [21,22,23,24,25]. A relevant example of piezoelectric devices is the macro-fiber composite (MFC), which consists of rectangular piezo-ceramic rods sandwiched between layers of adhesive films containing tiny electrodes [26,27]. The electrodes transfer a voltage directly to and from ribbon-shaped rods with thickness of a few tenths of a millimeter (Figure 1).

With reference to actuation capabilities, the MFC technology shows better performance and durability compared to other piezo-ceramic solutions, and can be used for both high and low frequency applications [28,29,30]. In the aerospace field, significant research efforts have been made on high frequency applications, with particular reference to vibration control [31,32] and wing ice protection systems [33]. In the low frequency domain, an interesting and challenging application is instead related to the so-called “morphing wings” (i.e., wing structures with the capability to dynamically modify their aerodynamic shape, to enhance efficiency, and/or to control the flight loads). Suitable and promising solutions were investigated by using MFC patches co-cured in or glued on substrates of composite material [34,35,36,37].

**Figure 2:**
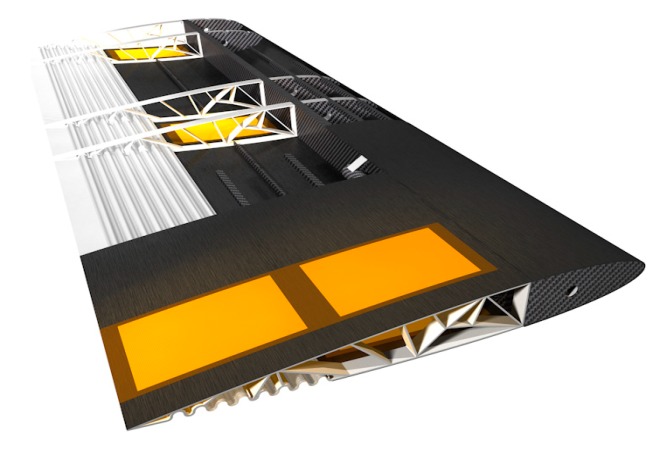
Example of integration of MFC patches in a wing structure [37].

MFC piezoelectric devices also have relevant potentialities as sensors, and a strong interest is growing in the aerospace field for their application in SHM or health usage and monitoring systems (HUMS). Relevant research efforts have been made for developing HUMS for aerospace structures with embedded, distributed, and miniature PZT devices, taking advantage from the capability of integration in complex geometries as well as to detect hidden damages [21,22,23,24,25,38,39,40]. In addition, special attention has been dedicated to the potential self-powered characteristics of these systems [41,42,43,44], bringing to promising SHM solutions with airplane structures integrating MFC patches [45] (Figure 2). Nevertheless, literature information and research results are currently poor if stand-alone devices are addressed.

This paper aims to provide a contribution within this research framework, with particular reference to the design of an electromechanical impedance-based SHM system [21,22,23,24] operating in different regimes. During flight, when normal loadings are applied, the patches work as energy harvesters by maintaining or restoring the battery charge of the stand-by SHM electronic board. If relevant or abnormal loadings occur, the patches instead act as sensors for the power-up SHM board, by acquiring in real-time relevant data on the component usage. Finally, during maintenance, the patches, operated by external high-voltage electronic equipment, can work as actuators, to stress the structure with pre-defined load profiles, as well as sensors, to monitor the structural response. It is worth noting that the vibration and/or loading levels strongly depend on the vehicle category (small or large airplane, turboprop or turbojet engines, helicopters, missiles, etc.) as well as on system location on the vehicle itself. Thus, the loading thresholds for the SHM board activation are expected to strongly depend on the reference application. Since this study is not focused on a particular aerospace case, the specifications/characteristics of loadings are not quantitatively given.

The work is articulated into three sections: in the first, the test rig used for the experimental characterization of the reference prototype is illustrated; successively, a model of the system dynamics is presented and validated against experimental data; finally, a model-based condition-monitoring algorithm is described and verified, by simulating different levels of system performance deviations.

## 2. Experimental Test System

**Figure 3:**
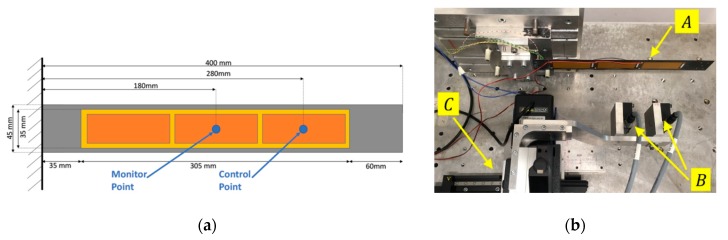
Experimental set-up [40]: (**a**) morphing laminate with MFC patches; (**b**) rig top view: MFC laminate (A); external laser sensors (B); precision translation stage (C).

As a reference structural element for the SHM studies, a carbon-epoxy composite laminate 400 × 45 × 2 mm × mm × mm is selected (Figure 3a and Table 1). Six MFC patches are installed and symmetrically arranged on the specimen (three per side), and the laminate is cantilevered to the rig frame. The patches are electrically connected and signaled to obtain a uniform distribution of bending torque along the laminate: the three patches on each side are connected in parallel and controlled by a dedicated channel, so that they are driven by opposite voltage levels.

**Figure 4:**
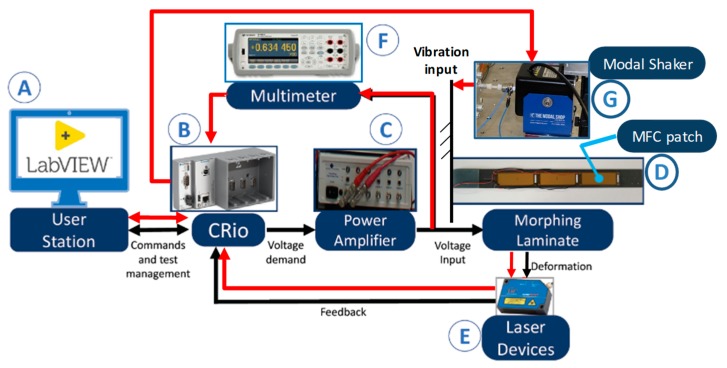
Experimental test system architecture.

The developed test system has two basic operating modes (Figure 4):

- Actuation mode (see black flows in Figure 4), in which the CRio embedded controller (B) receives from the user workstation (A) the demand voltages for the two channels of the morphing laminate, and a high-voltage amplifier (C) provides the MFC patches (D) with the electrical power;
- Sensing/energy harvesting mode (see red flows in Figure 4), in which the CRio embedded controller (B) receives from the user workstation (A) the demand vibration level for the modal shaker (G) connected to the laminate attachment, and a multimeter (F) measures the voltage generated by the MFC patches (D).

The rig is also equipped with two laser sensors (E, in Figure 4), measuring the laminate deflections at two points (Figure 3b) to supervise the deformation as well as to reconstruct the morphed shape. The measurement points on the laminate can be adjusted or modified by means of a precision translation stage installed on the rig (Figure 3b).

## 3. Development and Validation of the Model of System Dynamics

### 3.1. Dynamic Modelling

The results of a preliminary study carried out by the authors on the prototype [40] demonstrated that the system dynamics is characterized by well-separated behaviors in the low-frequency (<0.1 Hz) and medium/high-frequency (from 1 to 100 Hz) ranges. In particular, the low-frequency range is dominated by hysteresis, while at high frequencies the system’s response is characterized by structural resonances related to bending modes.

**Figure 5:**
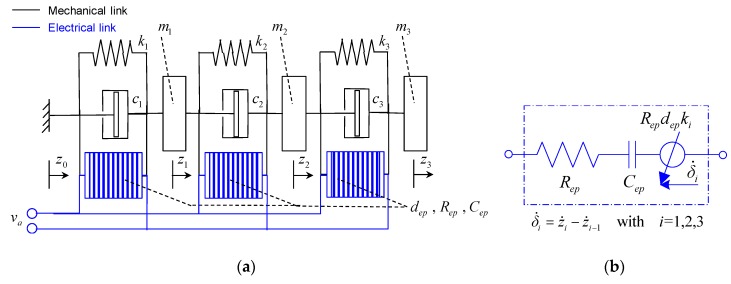
Lumped-parameters schematization: (**a**) complete prototype; (**b**) *i*-th patch.

For these reasons, by taking into account the electrical and structural symmetry of the prototype, the dynamic modelling was developed with reference to the lumped-parameters scheme, shown in Figure 5, to include:

- three bending vibration modes of the laminate;
- the charge dynamics of the three couples of MFC patches, taking account of the piezoelectric hysteresis.

Concerning the hysteresis modelling, several formulations are available in the literature to describe the phenomenon, and they are classified into two categories: static and dynamic models, depending on the use or not of ordinary differential equations (ODE) in the models themselves. Static models (e.g., Preisach [46], Prandtl-Ishlinskii [47], or polynomial models [48]) are typically very accurate, but they need a large number of experimental data for algebraic manipulations and interpolations, and their use for real-time condition monitoring is problematic [49]. On the other hand, dynamic models (e.g., Duhem [50], Maxwell [51], or Bouc-Wen [52,53] models) are often less accurate, but more adequate for control and real-time monitoring purposes. By selecting a Bouc-Wen hysteresis model, the system dynamics is described by the nonlinear twelfth-order ODE given by Equation (1),

(1) $$\left\{ \begin{array}{l} m_{3}\left({\ddot{\delta }}_{3}+{\ddot{\delta }}_{2}+{\ddot{\delta }}_{1}\right)=-c_{3}{\dot{\delta }}_{3}-k_{3}\delta_{3}+\frac{k_{3}d_{ep}}{C_{ep}}q_{h3}-m_{3}{\ddot{z}}_{0}\\ m_{2}\left({\ddot{\delta }}_{2}+{\ddot{\delta }}_{1}\right)=-c_{2}{\dot{\delta }}_{2}-k_{2}\delta_{2}+c_{3}{\dot{\delta }}_{3}+k_{3}\delta_{3}+\frac{k_{2}d_{ep}}{C_{ep}}q_{h2}-\frac{k_{3}d_{ep}}{C_{ep}}q_{h3}-m_{3}{\ddot{z}}_{0}\\ m_{2}{\ddot{\delta }}_{1}=-c_{1}{\dot{\delta }}_{1}-k_{1}\delta_{1}+c_{2}{\dot{\delta }}_{2}+k_{2}\delta_{2}+\frac{k_{1}d_{ep}}{C_{ep}}q_{h1}-\frac{k_{2}d_{ep}}{C_{ep}}q_{h2}-m_{3}{\ddot{z}}_{0}\\ \begin{array}{l} R_{ep}{\dot{q}}_{i}=-\alpha_{h}q_{i}/C_{ep}-R_{ep}k_{i}d_{ep}{\dot{\delta }}_{i}+v_{a}\\ {\dot{h}}_{qi}=A_{h}{\dot{q}}_{i}-\beta_{h}\left|{\dot{q}}_{i}\right|{\left|h_{qi}\right|}^{n-1}h_{qi}-\gamma_{h}{\dot{q}}_{i}{\left|h_{qi}\right|}^{n} \end{array}\;\mathrm{with}\;i=1,2,3 \end{array}\right.$$

where (with *i* = 1, 2, 3)

(2) $$q_{hi}=\alpha_{h}q_{i}+\left(1-\alpha_{h}\right)h_{qi}$$

(3) $$\delta_{i}=z_{i}-z_{i-1}$$

In Equations (1)–(3), ${\ddot{z}}_{0}$ is the base acceleration, *z_i_* and *δ_i_* are the displacement and the bending deformation of the *i*-th laminate section; *m_i_*, *k_i_*, and *c_i_* are the mass, the stiffness, and the structural damping of the *i*-th section of the laminate; *R_ep_*, *C_ep_*, and *d_ep_* are the equivalent resistance, capacitance, and piezoelectric coefficient of the patches; *A_h_*, *α_h_*, *β_h_*, *γ_h_*, and *n* are the Bouc-Wen model parameters; while *q_i_*, *h_qi_*, and *q_hi_* are the linear, the hysteretic, and the total charge of the *i*-th patch. Finally, *v_a_* depending on the operation regime of the system, represents the input voltage applied to the patches or the output voltage provided by them.

### 3.2. Experimental Validation of the Model

**Figure 6:**
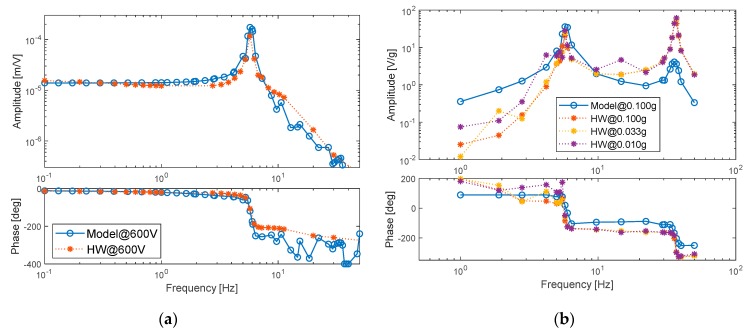
Model parameters’ tuning via frequency-domain responses: (**a**) deformation response to voltage input; (**b**) stored voltage response to base acceleration input.

**Figure 7:**
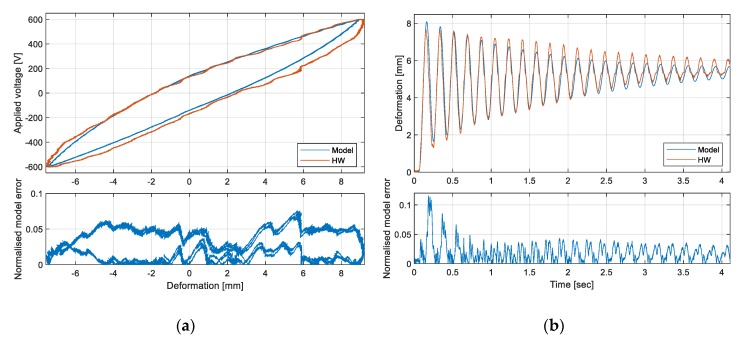
Validation of the model via time-domain responses: (**a**) hysteresis deformation loop with sinewave voltage input at 0.01 Hz; (**b**) deformation response to step voltage input of 300 V.

Aiming to collect the system identification database, the dynamics of the system prototype was experimentally characterized via an extensive test campaign, in terms of both time-domain and frequency-domain responses. The system response exhibited very good repeatability and the identification was developed in two main steps [54]:

- estimation of the model parameters (Table 2) by minimizing the model deviations from the hardware behavior with reference to sinusoidal frequency response tests, i.e.,
  ○ deformation responses to voltage inputs, with amplitudes from 150 to 600 V, ranging from 0.1 to 50 Hz, for a total of 120 tests and 200 simulations (Figure 6a);
  ○ stored voltage responses to acceleration inputs of amplitudes from 0.01 to 0.1 g, ranging from 1 to 50 Hz, for a total of 120 tests and 200 simulations (Figure 6b).
- model validation, by characterizing the model deviations from the hardware behavior with reference to time response tests, i.e.,
  ○ quasi-static deformation responses to high-amplitude/low-frequency sinewave voltage inputs (Figure 7a);
  ○ transient deformation responses to step voltage inputs (Figure 7b).

It is worth noting that the experimental investigation pointed out that the deformation response weakly depends on applied voltage amplitude, and no significant variation of the system dynamic behavior is observed by changing the input voltage amplitude. On the other hand, the stored voltage response strongly depends on base acceleration amplitude. For these reasons, Figure 6a reports the deformation response at 600 V amplitude only, while Figure 6b shows the voltage storage results for different acceleration amplitudes (note that also simulation results are amplitude-dependent, but only one acceleration amplitude is given in Figure 6b for the clarity of the plot).

Both experiments and simulations clearly point out that the resonant peak related to the first bending mode occurs at 5.8 Hz, the resistance-capacitance electric dynamics induces a real pole at about 10 Hz, while the second bending mode implies a resonance at 37 Hz. Experiments also point out the relevant voltage storage capability of the prototype, which is characterized by an acceleration-to-voltage gain that depends on vibration amplitude and achieves 35 V/g at 5.8 Hz (first resonance) and 68 V/g at 37 Hz (second resonance). The behavior is well reproduced by simulations, even if the prediction accuracy in terms of stored voltage amplitude is lower at the second resonance. This is probably due to that fact that the lumped-parameters model concentrates the laminate deformations at only three points, and this implies a decrease of accuracy at higher modes, in which the deformation distribution along the laminate is more complex.

Concerning the model validation, the activity is here focused on the actuation behavior only, essentially because the model-based condition-monitoring algorithm is designed via maintenance built-in test (MBIT) simulations, when the patches work as actuators. Validation of the model addressing the sensing/energy harvesting behavior will be a future development of this work.

Preliminary comparisons between model predictions and experimental data highlighted that the prediction error of a generic system variable (*y*) is linearly correlated to the amplitude of the variable itself, Equation (4):

(4) $$\left|y_{\mathrm{md}}-y_{\mathrm{hw}}\right|=e_{0y}+k_{erry}\left|y_{\mathrm{hw}}\right|$$

where *y*_md_ is the model prediction and *y*_hw_ is the hardware response, while *e*_0*y*_ and *k_err y_* are the bias and the gain characterizing the error on the variable *y*. For this reason, the evaluation of the prediction accuracy was made by normalizing the model error via Equation (5)

(5) $$\varepsilon_{my}=\frac{\left|y_{\mathrm{md}}-y_{\mathrm{hw}}\right|}{\left|y_{\mathrm{hw}}\right|+e_{0y}/k_{erry}}$$

Figure 7, related to deformation responses, actually demonstrates the effectiveness of the approach, since the normalized error does not significantly vary with the deformation amplitude for both quasi-static and fast transient tests. In the hysteresis test, the normalized deformation error is roughly constant during both discharge and charge phases, being 5% of the actual deformation in the charging phase and lower than 0.5% during discharge. Similar results are noted in the step response tests, where, after the initial transient, the mean value of the normalized error is 2% of the actual deformation, and its fluctuations are limited to 2%.

### 3.3. Assessment of the Energy Harvesting Capability

The experimentally-validated model of the prototype was used to assess its energy harvesting capability, aiming to support the feasibility of the self-powered system. The objective is to demonstrate that the MFC patches are capable of restoring the charge of a low-power battery, commonly used for supplying small electronic boards.

**Figure 8:**
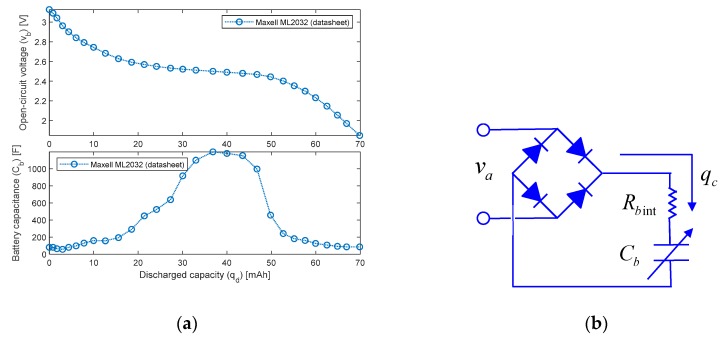
Energy harvesting simulation: (**a**) Maxell ML2032 Li-ion rechargeable battery characteristics [55]; (**b**) Thevenin battery model connected to the patches’ voltage output [56].

To perform the study, the open-circuit voltage characteristics (*v_b_*) of the 3V-65mAh Maxell ML2032 Li-ion rechargeable battery (Figure 8a, [55]) are used to calculate the equivalent capacitance (*C_b_*) of the Thevenin model of the battery (Figure 8b, [56]), Equation (6):

(6) $$C_{b}=-\frac{\partial q_{d}}{\partial v_{b}}\;\Rightarrow \;C_{b}=C_{b}\left(q_{d}\right)=C_{b}\left(v_{b}\right)$$

where *q_d_* is the discharged capacity of the battery in Amp∙sec.

Once defined, the maximum capacity of the battery (*q*_max_) and its initial charge (*q*_0_), the battery charge (*q_b_*) is expressed by Equation (7):

(7) $$q_{b}=q_{\max}-q_{d}=q_{\max}-q_{0}+q_{c}$$

and the battery charge dynamics obtained through the rectified MFC system voltage output (*v_a_*) is provided by Equation (8):

(8) $$v_{b}+R_{b\mathrm{int}}{\dot{q}}_{b}=\left|v_{a}\right|$$

By formulating the charge rate (${\dot{q}}_{b}$) as a function of the battery voltage and voltage rate,

(9) $$q_{b}=C_{b}\left(v_{b}\right)v_{b}\;\Rightarrow \;{\dot{q}}_{b}=\left(C_{b}+\frac{\partial C_{b}}{\partial v_{b}}v_{b}\right){\dot{v}}_{b}=\left(C_{b}-\frac{\partial^{2}q_{d}}{\partial v_{b}{}^{2}}v_{b}\right){\dot{v}}_{b}$$

we finally obtain the nonlinear first-order model describing the charging behavior:

(10) $${\dot{v}}_{b}=\frac{\left|v_{a}\right|-v_{b}}{R_{b\mathrm{int}}\left[C_{b}\left(v_{b}\right)-\frac{\partial^{2}q_{d}}{\partial v_{b}{}^{2}}v_{b}\right]}$$

**Figure 9:**
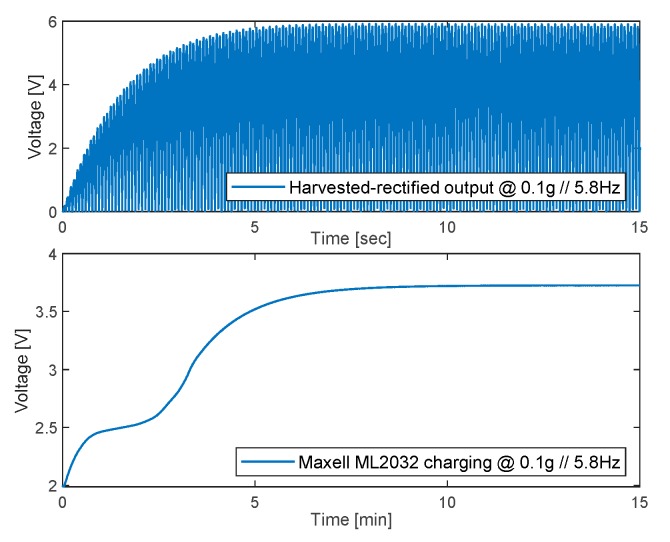
Energy harvesting simulation with sinusoidal base acceleration input (*R_b_*_int_ = 1 Ohm).

An example of the dynamics of the battery charge operated by the MFC patches is reported in Figure 9, where the simulation is carried out by imposing a 0.10 g sinusoidal base acceleration (${\ddot{z}}_{0}$ in Equations (1)–(3)) at 5.8 Hz (first resonance), and by assuming an internal battery resistance (*R_b_*_int_) of 1 Ohm. It is worth noting that the prototype system is capable of harvesting the energy coming from the mechanical vibrations, and to restore the battery charge within 10 min (starting from a completely discharged battery, i.e., 2 V).

## 4. System Condition Monitoring

The experimentally-validated model of the prototype was finally used to test a model-based condition-monitoring algorithm developed by the authors [40,57,58,59,60,61], in order to assess the system applicability to SHM systems. In model-based condition-monitoring approaches, the detection of faults or anomalies is actually performed by evaluating the deviation of hardware responses from the predictions of a system model related to normal operating conditions.

### 4.1. Condition-Monitoring Algorithm

**Figure 10:**
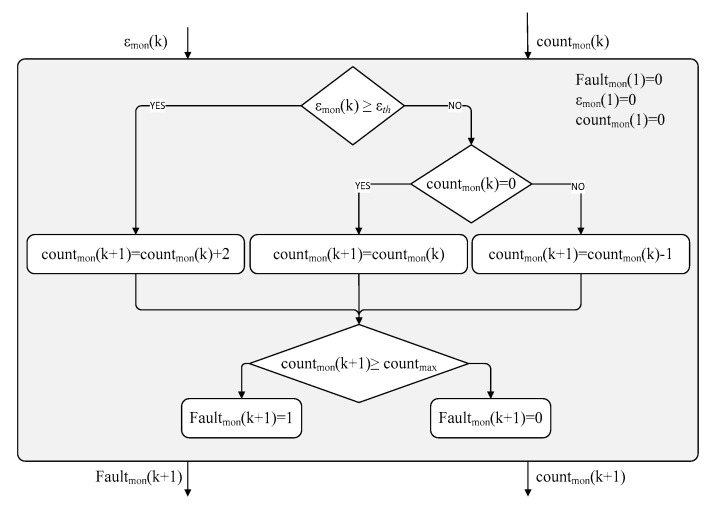
Flow chart of the condition-monitoring logic.

The proposed condition-monitoring logic, schematically reported in terms of flow chart in Figure 10, generates a fault flag (Fault_mon_, set to 1 in case of detection of detected anomalies) by elaborating the normalized model error (*ε*_mon_) sampled at the monitoring frequency (*f*_mon_). If the normalized error exceeds a threshold (*ε_th_*), a fault counter (count_mon_) is increased by 2; if the threshold is not exceeded, the fault counter is decreased by 1 when it is positive at the previous step, otherwise it is held to 0. The fault or anomaly is detected when the fault counter exceeds a pre-defined value (count_max_), a parameter which basically defines the fault latency of the algorithm.

In the present study, the monitored variable is the displacement of the laminate tip due to the bending deformation (*z*_3_ in Equations (1)–(3)), so the normalized model error is given by Equation (5), where *e*_0 *z*_ = 0.75 mm and *k_err_*_*z*_ = 0.075.

(11) $$\varepsilon_{\mathrm{mon}}=\frac{\left|z_{3\mathrm{md}}-z_{3\mathrm{hw}}\right|}{\left|z_{3\mathrm{hw}}\right|+e_{0z}/k_{errz}}$$

### 4.2. Maintenance Built-in-Test Simulations

The performances of the condition-monitoring algorithm are characterized by simulating the execution of two types of maintenance built-in tests (MBITs), i.e.,

- *MBIT.a*: response to sinewave voltage input at 0.1 Hz and 600 V (quasi-static test, Figure 7);
- *MBIT.b*: response to step voltage input with 300 V amplitude (fast transient test, Figure 7).

It is worth noting that, especially for the assessment of the transient response, several types of tests were studied [40], by varying both amplitude and waveform of the input signal, and the *MBIT.b* demonstrated a good balance between false alarms rejection and fault detection capability.

Once defined, the monitoring frequency (*f*_mon_), the error threshold (*ε_th_*), and the fault counter threshold (count_max_), the algorithm performances were characterized as follows:

*i*. for a subset of relevant system parameters (par), an acceptable range of deviations from the nominal behavior is defined. In this work, par **=** (*k_3_*, *α_h_*, *β_h_*), and this selection was made because the hysteresis parameters basically affect the low-frequency behavior, while the tip stiffness also impacts the high-frequency range, by varying the location of structural resonances;
*ii*. a model of the “degraded” hardware is obtained from the experimentally-validated model, by injecting uniformly-distributed random deviations on the selected model parameters;
*iii*. *MBIT.a* and *MBIT.b* are simulated by executing the condition-monitoring algorithms;
*iv*. steps *ii* and *iii* are repeated for *N* times, to cumulate a statistical database.

**Figure 11:**
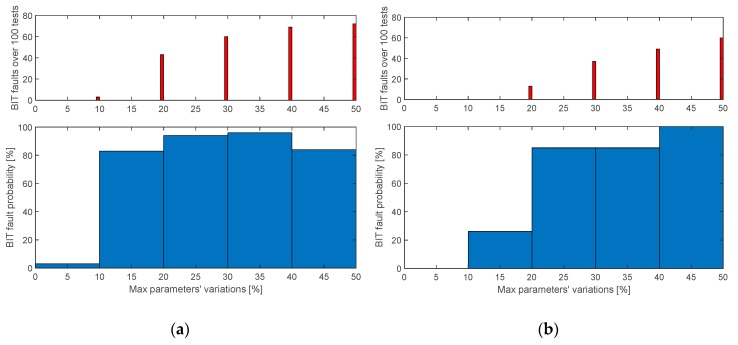
Performances of the condition-monitoring algorithm: (**a**) *MBIT.a*; (**b**) *MBIT.b*.

Figure 11 shows the results of the performance characterization of a condition-monitoring algorithm with the parameters reported in Table 3, by executing 100 simulations on each range of 10% deviations, in terms of number of detected BIT faults (upper plots) and BIT fault probability (lower plots) as functions of the deviation range.

Depending on the monitoring objectives, both *MBIT.a* and *MBIT.b* effectively support the maintenance. If the objective is to minimize the performance deviations, *MBIT.a* is more suitable, because its BIT fault probability is 83% for deviations from 10% to 20%, and it increases up to 94% for deviations from 20% to 30% (Figure 11a). On the other hand, if the objective is to accept limited performance deviations, *MBIT.b* is better because no BIT faults occur for deviations up to 10%, while the BIT fault probability is 85% for deviations from 20% to 40%, and 99% for deviations from 40% to 50% (Figure 11b). Clearly, the combined use of the two MBITs would enhance the entire process by minimizing false alarms and undetected anomalies. With particular reference to the false alarms rejection, it is worth noting that the monitoring performances were not characterized in the presence of measurements’ noise, because the noise impact on MBITs is expected to be minor (maintenance is performed in a controlled environment). On the other hand, the effects of noise are expected to be more relevant for in-flight monitoring algorithms (not addressed by this work).

## 5. Conclusions

A prototype of a self-powered SHM system made of a cantilevered composite laminate with six MFC patches was developed, and experimentally characterized in terms of actuation, sensing, and energy harvesting capabilities. A nonlinear dynamic model of the system, including piezoelectric hysteresis and three bending deformation modes, was developed starting from physical first principles, and validated with experiments. High fidelity was obtained in terms of deformation predictions (maximum normalized errors lower than 5% of the actual deformation), while the accuracy on stored voltage, though very good at the first resonance of the laminate (5.8 Hz), was lower at the second resonance (37 Hz). The results demonstrate the feasibility of a self-powered SHM system using the MFC technology, since the acceleration-to-voltage gain of the prototype is 35 V/g at the first resonance, and it achieves 68 V/g at the second resonance. The experimentally-validated model was then used to assess the energy harvesting capability of the prototype system, by demonstrating that the MFC patches are capable of restoring the charge of the 3V-65mAh Maxell ML2032 Li-ion rechargeable battery (commonly used for supplying small electronic boards). In particular, by imposing a 0.10 g sinusoidal base acceleration at 5.8 Hz, and assuming an internal battery resistance of 1 Ohm, the system is capable of harvesting the energy coming from the mechanical vibrations, and to restore the battery charge within 10 min (starting from complete discharge, i.e., 2 V).

Finally, the model is used to test a model-based condition-monitoring algorithm developed by the authors, by simulating two specifically-designed maintenance built-in tests. The “degraded” behavior was obtained from the “nominal” model, by injecting uniformly-distributed random deviations on relevant system parameters (the laminate stiffness and the hysteresis model coefficients), and 500 built-in-test simulations were performed. The results demonstrate that, with a combined use of the two proposed MBITs, the condition-monitoring algorithm effectively supports maintenance by checking the correspondence of performance to the nominal behavior as well as by detecting anomalies (BIT fault probability is 94% for deviations from 20% to 30%, and 99% for deviations from 40% to 50%).

In the paper, the model validation is focused on the actuation behavior, because the model-based condition-monitoring algorithm is designed via MBIT simulations when the patches work as actuators. Validation of the model addressing the sensing/energy harvesting behavior will be a future development of this work.

## Figures and Tables

**Table 1:** MFC patch operational parameters.

Parameter	Unit	Value
Voltage demand range	V	−500; 1500
Temperature operating range	°C	−20; 80
Operational frequency range	kHz	DC; 10
Stall force (tensile stress)	N	454
Free strain	ppm	1800
Lifetime @ 1 kV peak-to-peak	cycles	10^9^

**Table 2:** Dynamic model parameters (identification method legend: TM, test measurement; MEM, model error minimization; LA: literature assumption from [54]).

Symbol	Definition	Unit	Value	Identification Method
*R_ep_*	Patch resistance	Ohm	1500	TM
*C_ep_*	Patch capacitance	F	1.06 10^−5^	TM
*d_ep_*	Patch piezoelectric coefficient	m/V	1.37 10^−5^	MEM
*m*_1_	Mass of the root section of the laminate	kg	1.16 10^−3^	TM
*m*_2_	Mass of the intermediate section of the laminate	kg	2.32 10^−3^	TM
*m*_3_	Mass of the tip section of the laminate	kg	1.39 10^−3^	TM
*k*_1_	Stiffness related to root section bending	N/m	332.98	MEM
*k*_2_	Stiffness related to intermediate section bending	N/m	125.40	MEM
*k*_3_	Stiffness related to tip section bending	N/m	21.28	MEM
*c*_1_	Damping related to root section bending	N s/m	0.098	MEM
*c*_2_	Damping related to intermediate section bending	N s/m	0.020	MEM
*c*_3_	Damping related to tip section bending	N s/m	0.025	MEM
*α_h_*	Bouc-Wen linearity factor	--	0.605	MEM
*A_h_*	Bouc-Wen dissipation gain	--	−1	LA
*n*	Bouc-Wen shape factor	--	1	LA
*β_h_*	Bouc-Wen shape factor	V^−n^	8.307	MEM
*γ_h_*	Bouc-Wen shape factor	V^−n^	0	LA

**Table 3:** Condition-monitoring algorithm parameters.

Symbol	Definition	Unit	Value
*f*_mon_	Monitoring frequency	Hz	100
*ε_th_*	Normalized error threshold	--	0.225
count_max_	Fault counter threshold	--	200
